# 

**Published:** 1847-07

**Authors:** 


### CONTENTS

OF NO. I.

### ORIGINAL COMMUNICATIONS.

ESSAYS AND CASES.

ART. I.—Essay on the Endemic Summer Diseases of Floyd County, Indiana. By H. M. Dowling, M.D., of New Albany, Ia. - - - - - - - 1

ART. II.—Notes on Medical Matters and Medical Men in Paris. By DAVID W. YANDELL, M. D., of Louisville, Ky. - - - - - - - - - - - 19

ART. III.—New York Clinical Reports. By B. F. WENDEL, M.D., of Murfreesboro', Tennessee. - - - 42

BIBLIOGRAPHICAL NOTICES.

ART. IV.—New Elements of Operative Surgery. By ALPH. A. M. VELPEAU. Translated by P. S. TOWNSEND, M.D., late Physician to the Seamen's Retreat, Staten Island, New York, under the Supervision of, and with Notes and Observations by, VALENTINE MOTT, M.D., Professor of Operative Surgery in the University of New York. Vol. III. - - - - - - - - 57

ART. V.—A Treatise on Diseases of the Eye. By W. LAWRENCE, F.R.S. A new edition, edited, with numerous Additions, by ISAAC HAYS, M.D., Philadelphia. - 58

ART. VI.—Medical Botany; or, Descriptions of the more important Plants used in Medicine, with their History, Properties, and Mode of Administration. By R. E. GRIFFITH, M.D., Philadelphia. - - - - - 58

ART. VII.—The Virginia Springs, with their Analysis, and some Remarks on their Character; together with a Directory for the Use of the White Sulphur Water, and Account of the diseases to which it is Applicable. By JOHN J. MOORMAN, M.D., Resident Physician at the White Sulphur Springs. - - - - - - 60

ART. VIII.—The Medical Student's Vade-Mecum. By G. MENDENHALL, M.D., Philadelphia. - [illegible]2

SELECTIONS FROM AMERICAN AND FOREIGN JOURNALS.

Diseases from the Immoderate Use of Tobacco. - - 63
Medical Character of Saratoga Springs. - - - - 66
Ether Vapor in the Practice of Midwifery. - - - 70
Inhalation of Ether in Instrumental Labor. - - - 71
Injurious Symptoms from the Inhalation of Ether Vapor. - 78
Croup Cured by Cauterizing the Larynx with Nit. Arg. - 81
Medical Convention.—Report on a Uniform Standard of Requirements for the Degree of M.D. - - - - 82

ORIGINAL INTELLIGENCE.

National Medical Convention. - - - - - - - - 88
Resignation of Prof. Hare. - - - - - - - - - 89
Veterinary Medicine. - - - - - - - - - 90
Death of Prof. Warner. - - - - - - - - - 90
Death of Lisfranc. - - - - - - - - - - 90
Painless Operation. - - - - - - - - - - 91
Temperature of Kenhawa Salt Wells. - - - - - 92

